# Modelling the outcomes of different red blood cell transfusion strategies for the treatment of traumatic haemorrhage in the prehospital setting in the United Kingdom

**DOI:** 10.1111/vox.13359

**Published:** 2022-09-14

**Authors:** Barnaby Roberts, Laura Green, Venus Ahmed, Tom Latham, Peter O'Boyle, Mark H. Yazer, Rebecca Cardigan

**Affiliations:** ^1^ Department of Health and Social Care Health Protection Analysis London UK; ^2^ Blizard Institute Queen Mary University of London London UK; ^3^ NHS Blood and Transplant London UK; ^4^ Barts Health NHS Trust London UK; ^5^ Department of Pathology University of Pittsburgh Pittsburgh Pennsylvania USA; ^6^ Department of Haematology University of Cambridge Cambridge UK

**Keywords:** blood transfusion, prehospital setting, quality‐adjusted life years, trauma

## Abstract

**Background and Objectives:**

The limited supply and increasing demand of group O RhD‐negative red blood cells (RBCs) have resulted in other transfusion strategies being explored by blood services that carry potential risks but may still provide an overall benefit to patients. Our aim was to analyse the potential economic benefits of prehospital transfusion (PHT) against no PHT.

**Materials and Methods:**

The impact of three PHT strategies (RhD‐negative RBC, RhD‐positive RBC and no transfusion) on quality‐adjusted‐life‐years (QALYs) of all United Kingdom trauma patients in a given year and the subset of patients considered most at risk (RhD‐negative females <50 years old), was modelled.

**Results:**

For the entire cohort and the subset of patients, transfusing RhD‐negative RBCs generated the most QALYs (141,899 and 2977, respectively), followed by the RhD‐positive RBCs (141,879.8 and 2958.8 respectively), and no prehospital RBCs (119,285 and 2503 respectively). The QALY difference between RhD‐negative and RhD‐positive policies was smaller (19.2, both cohorts) than RhD‐positive and no RBCs policies in QALYs term (22,600 all cohort, 470 for a subset), indicating that harms from transfusing RhD‐positive RBCs are lower than harms associated with no RBC transfusion. A survival increase from PHT of 0.02% (entire cohort) and 0.7% (subset cohort) would still make the RhD‐positive strategy better in QALYs terms than no PHT.

**Conclusion:**

While the use of RhD‐positive RBCs carries risks, the benefits measured in QALYs are higher than if no PHT are administered, even for women of childbearing potential. Group O RhD‐positive RBCs could be considered when there is a national shortage of RhD‐negative RBCs.

**Highlights**

- The difference in quality‐adjusted life‐years (QALYs) between group O RhD‐negative and O RhD‐positive red cell transfusion was much smaller than that between O RhD‐positive and no prehospital transfusion (PHT).
- The benefits of transfusing group O RhD‐positive red cells, measured in terms of QALYs, are substantially greater than if no prehospital transfusion is administered, even for RhD‐negative women of childbearing potential.

## INTRODUCTION

Several observational studies have demonstrated survival benefits following the administration of red blood cells (RBCs) or low titre group O whole blood (LTOWB, herein referred to as RBCs) compared to saline alone in the resuscitation of traumatic haemorrhagic patients in the prehospital environment [1, 2, 3]. Randomized controlled trials (RCTs) that have compared prehospital blood transfusion to crystalloids have shown different outcomes due to differences in the patients' underlying demographics and clinical environments in which the studies were conducted. An RCT in which the standard of prehospital care was supplemented with two units of plasma for trauma patients transported to the hospital by helicopter showed that plasma transfusion significantly improved 30‐day survival by approximately 10% [4], with the secondary analysis showing that the combination of RBC and plasma produced the highest survival rates compared to not providing any blood products while *en route* to the hospital [5]. A recent RCT (RePHILL trial) showed that RBC transfusion plus lyophilized plasma in the prehospital setting was not superior to saline resuscitation for improving tissue perfusion or reducing episode mortality; however, at 24 h, the adjusted average differences in mortality were 7% lower in the blood components arm (personal communication from trial investigators) [6].

In the prehospital setting, group O RhD‐negative RBC is transfused to avoid harms caused by the RhD‐positive blood being transfused to RhD‐negative recipients because the recipient's ABO/RhD type is known. These harms include severe haemolysis and possibly death [7, 8], a haemolytic transfusion reaction (HTR) following the transfusion of an RhD‐positive RBC unit to a recipient with preformed anti‐D [9, 10], and for the RhD‐negative female of childbearing potential (FCPs) who has become D‐alloimmunized through RhD‐positive blood, this antibody can cause haemolytic disease of the foetus and newborn (HDFN) should she later become pregnant.

However, the ability to provide RhD‐negative RBCs for prehospital transfusion (PHT) is constrained by donor supply. The current demand for group O RhD‐negative RBCs in England is 13% of the supply compared to the 7% frequency of group O RhD‐negative in the general population. An international study of blood centre collectors found that only 10% of their RBC distributions to hospitals were group O‐negative [11]. Therefore, the supply of these precious products is very limited. Several studies have modelled the overall clinical risk of foetal/neonatal outcomes following the transfusion of RhD‐positive RBCs to injured RhD‐negative recipients and FCPs [12, 13, 14, 15]. However, evaluation of the risk–benefit ratio of providing group O RhD‐positive RBCs to trauma patients in terms of quality‐adjusted‐life‐years (QALYs) gained has not been evaluated before.

In this study, we compare the relative advantages of different policies for providing PHT, whereby the effect on the recipient's QALY was modelled in the following three scenarios: using RhD‐negative RBCs for all PHTs, using RhD‐positive RBCs for all PHTs, or not administering PHTs to injured patients.

## METHODS

We modelled the impact of three scenarios on two groups of trauma patients: (i) a representative sample of all United Kingdom trauma patients in a given year, and (ii) the subset of patients who were RhD‐negative females of childbearing potential <50 years old (referred as FCP), by simulating the harms experienced by patients over the remainder of their lifetimes. In the case of HDFN occurrence, we also modelled its impact on the affected babies.

A cohort was defined as a representative sample of patients who suffered a trauma in the United Kingdom in 1 year (5561 patients), as calculated previously [16]. For post‐trauma life expectancy, we used the Office for National Statistics data and set this to 48 years after the accident for the entire cohort and 56 years after the accident for the subset cohort of FCPs due to their lower age at the time of injury, accounting for the average age of trauma patient and the impact of the trauma on life expectancy [17].

We modelled the reductions in health‐related quality of life (HRQoL) following the trauma using a health economics approach to capture the QALYs associated with each transfusion scenario. To calculate QALYs associated with each transfusion scenario, we obtained the HRQoL associated with each post‐trauma outcome and modelled the number of years patients would spend in different health states to attain the QALYs experienced. HRQoL evidence was obtained by conducting a systematic literature review to find studies using EQ‐5D [18], SF‐36 [19] and HUI‐3 [20] stated preference questionnaires.

### Modelling impact on patients suffering trauma

**FIGURE 1:**
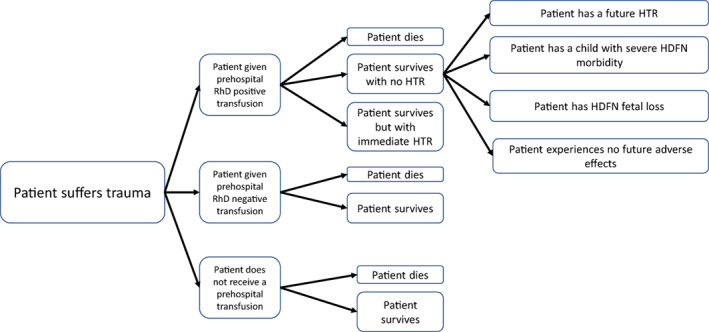
Pathway of possible patient outcomes following trauma and PHT. HDFN, haemolytic disease of the foetus and newborn; HTR, haemolytic transfusion reaction

For each three resuscitation scenarios (prehospital RhD‐positive RBC transfusion, or RhD‐negative RBC transfusion, or no PHT) and the two patient groups (overall cohort and FCPs), we ‘allocated’ patients to six health states in each year following transfusion (Figure 1):

1. Patient survives trauma without harm
2. Patient dies following trauma
3. Patient survives trauma but experiences HTR morbidity
4. Patient survives trauma but dies from HTR
5. Patient survives trauma but has a child with severe HDFN morbidity
6. Patient survives trauma but has a child with HDFN mortality

Allocations 3–6 were only relevant when RhD‐positive blood was transfused, and the recipient was RhD‐negative. The probability of trauma survival and death was based on values obtained from two studies [1, 2] demonstrating survival benefits following PHT of injured patients. The probability of suffering transfusion harms was based on a previously published model [15]. Key inputs used for the model are provided in Table A1 in Supporting information. The RePHILL trial performed in England showed that prehospital RBC transfusion plus LyoPlas was not superior to saline resuscitation in reducing the composite outcome of episode mortality and lactate clearance in trauma bleeding patients [6]. However, the study's design and findings have been criticized, including the selection of the mortality time point in the primary outcome, the length of time elapsed from injury until the administration of the study intervention, the highly injured nature of the patients and their correspondingly high death rate [21, 22]. There was, however, a 7% absolute risk reduction (25% relative risk reduction) among the PHT recipients at 3 h compared to those who were resuscitated with saline. Thus, the sensitivity analysis was designed to include this risk reduction.

Where a patient was predicted to have a future child with severe HDFN morbidity due to RhD‐positive RBCs during the resuscitation, we modelled a QALY impact to both the patient and child, where the impact to the patient is based on the impact to HRQoL observed for mothers of children with Cerebral Palsy, as there were no other published data on the impact of HRQoL for mothers of children with HDFN [23, 24, 25]. For patients that experience foetal death due to HDFN caused by anti‐D that was formed following receipt of RhD‐positive RBCs during trauma resuscitation, we only modelled a QALY impact on the child based on studies that showed that there was no HRQoL impact to these mothers [26]. In our sensitivity scenarios we considered the impact on the mothers of foetal death.

We calculated the impact of PHTs/no transfusions over the whole of one cohort's lifetime and expressed this in two sets of outcome measures (Figure 1). The first set of outcome measures was split by health state and included: (i) the number of patients ending up in each health state; (ii) the total QALYs for all patients in each health state in a given year, and; (iii) the discounted monetized value of QALYs for each health state. These enabled us to calculate a second set of aggregated measures: (i) the summed total of QALYs across all health states; (ii) the summed value of discounted monetized QALYs across all health states, and; (iii) the summed total of QALYs across all health states per person.

### Modelling impact on babies born to patients who suffered trauma

When RhD‐positive RBCs were transfused, we calculated the potential impact on babies born with HDFN or on those who died because of HDFN. Whereas the specific harms to patients were expressed as the QALYs attributed to patients in each health state, the harms to babies dying from HDFN or experiencing a long‐term adverse event from it were expressed as the QALYs lost, compared to if those babies had not suffered HDFN. This QALY difference was subtracted from the total QALYs experienced by the cohort. Key Modelling Assumptions made are described under Supporting information.

### Sensitivity analysis

The sensitivity analysis modified three sets of parameters to test ‘worst‐case’ scenarios: (i) HRQoL measures, (ii) probabilities of harms leading to the three risks, and (iii) trauma survival rates. Overall, five scenarios were modelled for each policy option: the main scenario, three sensitivity scenarios changing the parameter groups above individually, and one sensitivity scenario changing all parameters simultaneously.

## RESULTS

Based on survival data from studies of patients who received PHTs [1, 2], we calculated that an estimated 5561 injured patients per year in England who received PHTs, 634/5561 (11.4%) would die within the first 24 h. Patients who received RhD‐negative RBCs would not have experienced immediate or delayed HTRs caused by anti‐D or be at risk for future HDFN. The total number of QALYs over the lifetime of the recipients of prehospital RhD‐negative RBCs who survived the trauma was calculated to be 141,899 (Table 1).

**TABLE 1:** Outcomes of the entire cohort of injured patients (*n* = 5561) stratified by the RhD‐type of prehospital RBCs transfused

Outcomes	Prehospital RhD‐negative RBC recipients			Prehospital RhD‐positive RBC recipients			No prehospital RBC transfusion		
	No. of patients	Total QALY per person over lifetime	Total QALYs in entire population	No. of patients	Total QALY per person over lifetime	Total QALYs in entire population	No. of patients	Total QALY per person over lifetime	Total QALYs in entire population
Patient survives trauma without harm	4927	28.8	141,899	4926.5	28.8	141,883	4360	27.4	119,285
Patient dies in trauma	634	0	0	634	0	0	1201	0	0
Patient survives trauma but experiences HTR morbidity	0	0	0	0.19	28.7	5	0	0	0
Patient survives trauma but dies from HTR	0	0	0	0.02	0	0	0	0	0
Patient survives trauma but has a child with severe HDFN morbidity	0	0	0	0.13	17.8	3	0	0	0
Patient survives trauma but has a child with HDFN mortality	0	0	0	0.18	28.8	5	0	0	0
Foetus/neonate experiences major morbidity from HDFN	0	0	0	0.13	NA	−4.8	0	0	0
Foetus/neonate dies from HDFN	0	0	0	0.18	NA	−12.4	0	0	0
Total	5561		141,899	5561		141,879.8	5561		119,285
Discounted QALYs			102,147			102,135			85,868
Discounted QALYs per patient			18.368			18.367			15.441
Discounted monetized QALYs			£7,150,296,980			£7,149,599,392			£6,010,768,839
Discounted monetized QALYs per patient			£1,285,793			£1,285,668			£1,080,879

Abbreviations: HDFN, haemolytic disease of the foetus and newborn; HTR, haemolytic transfusion reaction; QALY, quality‐adjusted life year; RBC, red blood cell.

For patients who received RhD‐positive RBCs, the same percentage would be expected to die at 24 h because both RhD‐positive and RhD‐negative RBCs were assumed to confer the same survival benefits. However, due to receipt of RhD‐positive RBCs, the model predicted that, of these survivors, there would be a small number of patients who would directly experience any anti‐D mediated adverse events. In addition, the receipt of the RhD‐positive RBCs would be expected to cause 0.13 foetal/neonatal major morbidity events and 0.18 foetal/neonatal mortality events due to HDFN caused by transfusing RhD‐positive RBCs to the female trauma patients in this cohort. These foetal/neonatal adverse events reduce the total number of QALYs among the recipients of RhD‐positive prehospital RBCs to 141,879.8, a difference of 19.2 QALYs for the entire cohort compared to recipients of prehospital RhD‐negative RBCs, with the monetization value being almost £600,000.

For the group of patients who did not receive a PHT, the model predicted that there would be 1201/5561 (21.6%) deaths leading to approximately 22,600 fewer QALYs over the lifetime of the recipients in this group (119,285) compared to those who received a PHT. There were no other anti‐D mediated adverse events predicted to occur in this group, as they were not exposed to the RhD‐antigen through PHT. Using the valuation approach outlined in the Green Book [27], a publication from the United Kingdom Treasury that provides guidance on how to appraise policies, programmes and projects, the resulting loss of QALYs from not providing RBCs for prehospital resuscitation would be valued at just over £1billion compared to providing RBCs of any RhD type over the lifetime of these patients (Table 1).

Of the 5561 patients in England who received a PHT, 100 would have been FCPs who were at risk of a future pregnancy affected by HDFN [15]. The provision of RhD‐positive RBCs would be expected to lead to very few patients in this cohort experiencing any of the anti‐D mediated adverse events, and there would be 0.13 foetal/neonatal major morbidity events and 0.17 foetal/neonatal mortality events per year due to HDFN caused by transfusing RhD‐positive RBCs to this group. Because of the small number of adverse events caused by using RhD‐positive RBCs, the total number of QALYs among these recipients would be reduced by only 18.2 compared to the group that received RhD‐negative RBCs (2977 vs. 2958.8, respectively). Not providing RBCs for prehospital resuscitation would diminish the total number of QALYs for FCPs by approximately 470 compared to RBC recipients due to the smaller number of expected survivors among the non‐transfused patients. For these FCPs, not providing prehospital RBCs would result in a QALY loss worth approximately £22.7 million compared to providing RBCs of any RhD type over the lifetime of these patients (Table 2).

**TABLE 2:** Outcomes of the subset cohort of injured women <50 years old (*n* = 100) stratified by the RhD‐type of prehospital RBCs transfused

Outcome	Prehospital RhD‐negative RBC recipients			Prehospital RhD‐positive RBC recipients			No prehospital RBC transfusion		
	No. of patients	Total QALY per person over lifetime	Total QALYs in entire population	No. of patients	Total QALY per person over lifetime	Total QALYs in entire population	No. of patients	Total QALY per person over lifetime	Total QALYs in entire population
Patient survives trauma without harm	88.6	33.6	2977	88.3	33.6	2966	78.4	31.9	2503
Patient dies in trauma	11.4	0	0	11.4	0	0	21.6	0	0
Patient survives trauma but experiences HTR morbidity	0	0	0	0.01	33.5	1	0	0	0
Patient survives trauma but has a child with severe HDFN morbidity	0	0	0	0.001	0	0	0	0	0
Patient survives trauma but has a child with HDFN mortality	0	0	0	0.13	20.7	3	0	0	0
Foetus/neonate dies from HDFN	0	0	0	0.17	33.6	6	0	0	0
Foetus/neonate experiences major morbidity from HDFN	0	0	0	0.13	NA	−4.9	0	0	0
Foetus/neonate dies from HDFN	0	0	0	0.17	NA	−12.3	0	0	0
Total	100		2977	5561		2958.8	100		2503
Discounted QALYs			2035			2025			1710
Discounted QALYs per patient			20.345			20.254			17.103
Discounted monetized QALYs			£142,415,783			£141,778,854			£119,719,272
Discounted monetized QALYs per patient			£1,424,158			£1,417,789			£1,197,193

Abbreviations: HDFN, haemolytic disease of the foetus and newborn; HTR, haemolytic transfusion reaction; QALY, quality‐adjusted life year; RBC, red blood cell.

### Sensitivity analyses

Sensitivity analyses, given a degree of uncertainty relating to some of the key model inputs, were performed by adjusting some of these parameters (Table 3). Changing the HRQoL parameters caused a reduction of only four QALYs in the overall (0.00%) and the subset (0.14%) cohorts when RhD‐positive blood was used. This is mainly from considering an HRQoL impact on a mother experiencing foetal death which was not considered in the main scenario. Similarly, the difference in the total number of lifetime QALYs for the probability of experiencing an adverse event between the main and the sensitivity analyses of the overall cohort was very small (0.05%; 67 QALYs), with a slightly higher difference (1.67%; 49 QALYs) observed in the analysis of the subset of FCP.

**TABLE 3:** Sensitivity analysis performed on the entire cohort of injured patients (*n* = 5561) and on the subset cohort of injured women <50 years old (*n* = 667)

	Overall cohort (*n* = 5561)				Subset analysis of women ≤50 (*n* = 100)			
	Main analysis	Sensitivity analysis	Total lifetime QALYs whole population—main​	Total lifetime QALYs whole population—sensitivity​	Main analysis	Sensitivity analysis	Total lifetime QALYs whole population—main​	Total lifetime QALYs whole population—sensitivity​
(A) QoL parameter								
QoL of child post HDFN	0.56	0.47			0.56	0.47		
QoL of mother following foetus/neonatal HDFN death	0.6	0.3			0.6	0.3		
QoL of mother of HDFN child with disabilities	0.37	0.3			0.37	0.3		
*Total*			141,880	141,876			2958	2954
(B) Harms' probabilities								
Probability of immediate HTR	0.004%	0.025%			0.016%	0.12%		
Probability of future HTR	0.00021%	0.0006%			0.0014%	0.024%		
Average number of babies per (surviving) patient	0.10	0.14			0.81	0.99		
Probability that baby is at risk of HDFN	2.34%	4.9600%			15.60%	31.02%		
Probability that an at‐risk baby dies from HDFN	0.015%	0.023%			0.015%	0.023%		
Probability that an at‐risk baby has severe disability from HDFN	0.012%	0.017%			0.012%	0.02%		
*Total*			141,880	141,813			2958	2909
(C) 24‐h survival rate (%)								
Prehospital RhD −ve RBC transfusion	88.6%	81.4%	141,899	130,368	88.6%	81.4%	2977	2735
Prehospital RhD +ve RBC transfusion	88.6%	81.4%	141,880	130,350	88.6%	81.4%	2958	2718
No PHT	78.4%	78.4%	119,285	119,285	78.4%	78.4%	2503	2503
All sensitivities changed								
Prehospital RhD −ve RBC transfusion				130,368				2735
Prehospital RhD +ve RBC transfusion				130,274				2659
No PHT				119,285				2503

*Note*: The main analysis refers to the analysis that was performed using the values described in the methods; the sensitivity analysis refers to the analysis performed using the values listed for the three parameters in this table.

Abbreviations: HDFN, haemolytic disease of the foetus and newborn; HRQoL, health‐related quality of life; HTR, haemolytic transfusion reaction; QALY, quality‐adjusted life year; RBC, red blood cell.

The third sensitivity scenario reduced the survival benefits from 10.2% to 3% (compared to no transfusion), based on the effect of PHTs on episode mortality observed in the RePHILL trial [6]. With lower survival rates, fewer QALYs were gained regardless of the RhD nature of the transfused blood. However, even with reduced survival benefits, prehospital RBC transfusion of either RhD‐type continued to be advantageous compared to no transfusion. Survival increases of only 0.02% for the entire cohort and 0.7% for the subset cohort from providing PHTs would be sufficient to make an RhD‐positive strategy better in terms of QALYs than not providing PHTs. Changing all sensitivity parameters simultaneously still showed that prehospital RBC transfusion offers greater QALYs gained compared to not providing PHTs, even if RhD‐positive blood is used.

## DISCUSSION

Previous models of transfusing RhD‐positive RBCs to injured trauma patients of unknown RhD‐type have presented the risks of adverse events as probabilities of their occurrence [12, 13, 14]. This study is unique because it quantified the relative benefit of PHT in terms of QALYs gained, which provides another means of comparing different approaches to implementing a PHT program. Our results show that while the use of RhD‐positive RBCs carries small risks, the benefits measured in QALYs are substantially larger than if no PHTs are administered, even for RhD‐negative FCPs. This suggests that if RhD‐negative RBCs are unavailable, the use of RhD‐positive RBCs is a preferable alternative to not providing any prehospital RBC transfusions.

This result is driven by the survival benefits of providing PHTs, while the likelihood of RhD‐positive RBCs causing harm is comparatively very low. In our model, we assumed a survival benefit of 10.2% based on two observational studies [1, 2]; however, there is uncertainty about the magnitude of survival benefit from providing PHTs. The sensitivity analysis of the model demonstrated that even when we lowered the survival benefit to 3% in line with episode mortality findings from the RePHILL trial [6]—RhD‐positive RBCs are preferable to not providing a PHT.

Following the United Kingdom government Treasury green book [27] approach for valuing QALYs, a PHT provides approximately £205,000 worth of value per patient on average, that is, the difference in discounted monetized QALYs per patient receiving RhD‐positive or negative blood compared with no transfusion. The NHS cost of supplying a unit of RBCs is £153.10. The cost of administering an RBC transfusion is estimated as £57 for the first unit and £36 for subsequent units [28]. Therefore, the cost for a patient receiving two units of RBCs prehospital, which is the current average amount transfused in the prehospital setting from a recent survey of emergency medical services in the United Kingdom (LG personal communication), is about £400. This is substantially lower than the value of the QALY benefit. PHTs provide 2.93 discounted QALYs per patient, which at the cost of £400, is the cost per QALY of £137 and is substantially below the NICE guide of £20,000–30,000 per QALY.

Comparing the discounted monetized QALYs per patient between recipients of RhD‐negative RBCs and RhD‐positive RBCs, the use of RhD‐negative blood provides about £125 more value per patient than RhD‐positive blood. However, the equivalent figure for RhD‐negative FCPs is £6369, indicating that a policy of providing RhD‐negative blood for FCPs provides benefits and should be pursued where possible. Hospitals in England currently pay the same amount for RhD‐negative and RhD‐positive group O blood. However, RhD‐negative blood is in higher demand, and blood services spend additional resources to recruit and retain more RhD‐negative donors. The totality of these costs is not well described in the literature, but it seems unlikely to exceed the value of providing RhD‐negative blood to FCPs. Furthermore, it is less clear what the totality of these costs will be for the whole patient group and whether recruitment costs will outweigh patient benefits, and further research is required to understand these.

This study has several limitations. First, since the model was built using data from the literature, the extent to which these findings can be generalizable depends on the generalizability of the original data. Secondly, it would have been good to have provided a projection on the effect of blood transfusion on QALYs based on the patient's injury severity; however, all studies/trials have pooled the transfusion effect for all types and injury severity, and we were not able to quantify this in QALY terms. Thirdly, this study was designed to describe the effect of PHT on the 24‐h mortality rate, as this time‐point is the most likely to be modifiable by PHT, as beyond the 24 h, the cause of death in trauma patients is typically factors other than exsanguination. Indeed, the American National Heart, Lung, and Blood Institute and Department of Defence have recently supported the development of short‐term outcomes (3–6 h mortality) for adult trauma patients based on recent RCTs [29]. In the United Kingdom, the RCT of whole blood versus standard of care in the prehospital setting, will use the composite of mortality or massive transfusion at 24 h as its primary outcome. Therefore, the use of 24 h mortality in this model took all these development into consideration.

In conclusion, this study quantified the change in QALYs based on two PHT strategies compared to not providing PHTs to injured patients. While a small number of patients were predicted to experience harm from receiving RhD‐positive RBCs, the number of QALYs gained by providing prehospital RBCs was much larger. Our data support a policy of using RhD‐negative blood for FCPs where possible. The quantification of transfusion risks and benefits prehospital in terms of QALY also enables future work to compare the allocation of group O RhD‐negative blood between different clinical settings to maximize health outcomes and to consider cost‐effectiveness.

## CONFLICT OF INTEREST

R.C. is a member of Terumo BCT's Physician Advisory Board; L.G. and M.Y. have received expenses for speaking at conferences sponsored by Terumo BCT. L.G. has received unrestricted research grants from Barkey Plasmatherm.

## Supporting information


**Appendix S1.** Supporting Information.
