# Supplementary material for: Modelling the outcomes of different red blood cell transfusion strategies for the treatment of traumatic haemorrhage in the prehospital setting in the United Kingdom
Source: Vox Sang. 2022 Sep 14;117(11):1287–95. doi: 10.1111/vox.13359 (PMC9825834; doi:10.1111/vox.13359)
Supplement: Supplementary file 1 — Appendix S1. Supporting Information. [file VOX-117-1287-s001.docx]

**Supplemental material**

*Key Modelling Assumptions*

We assumed the following in each aspect of the modelling process:

1. *Probabilities*: The risk of immediate/future HTR and HDFN harms (morbidity or mortality) were assigned different probabilities based on whether they occurred: (i) immediately or in the future, and; (ii) if morbidity or mortality occurred. The risk of future HTR was assumed to be the same across years of a cohort’s lifetime. For simplicity, HDFN harms were modelled to have equal chance of occurring in each year until a female patient turns 50, so that the modelled number of children was equivalent to the expected number of children for a recipient of the average trauma age and sex.
2. *Health-Related Quality of Life*: The change in HRQoL for a patient or baby associated with an HDFN harm lasted for the remainder of their life. The change in HRQoL for those suffering HTRs lasted for one year only before reverting to the ‘healthy’ HRQoL value. Trauma patients’ HRQoL was downgraded after trauma (based on available literature)^1,2^ and this differed between the ‘no transfusion’ and ‘transfusion’ groups.^1,3^ We modeled HRQoL reductions to mothers of children with severe HDFN morbidity, but not if the fetus died from HDFN.
3. *Life Expectancy*: This was assumed to be the average remaining life expectancy of someone in the UK population after controlling for cohort characteristics, minus life expectancy lost to trauma survivors.^1^ The life expectancy of babies suffering from HDFN was modelled as being lower than the UK average.^4,5^
4. *Harms’ Conceptualization*: Patients only ever suffer one harm (meaning also that mothers only birth one baby with a harm), since the rate of harms is calculated as an average over the population.
5. *Health Economics*: QALYs are valued at the Department of Health and Social Care (DHSC) standard rate of £70,000: prior to April 1^st^ 2022, the value was £60,000. The discount rate used when QALYs are monetized is 1.5%, as per DHSC guidance for discounting health benefits.^6^

**Table A.** Data inputs for the R-modelling.

HRQoL = Health related quality of life. HTR = hemolytic transfusion reaction. HDFN = hemolytic disease of the fetus and newborn. RBC = red blood cell. N/A – not applicable.

|  | **Overall cohort (n = 5561)** | | |
| --- | --- | --- | --- |
|  | **No RBC Blood** | **RhD negative RBC** | **RhD positive RBC** |
| **Survival rate** | 0.78 | 0.89 | 0.89 |
| **HRQoL no harm** | 0.57 | 0.6 | 0.6 |
| **HRQoL Mother of HDFN child – severe disability** | N/A | N/A | 0.37 |
| **QoL Child with HDFN – severe disability** | N/A | N/A | 0.56 |
| **Reduction in QoL due to HTR** | N/A | N/A | 0.05 |
| **Probability of immediate HTR** | N/A | N/A | 0.004% |
| **Probability of future HTR** | N/A | N/A | 0.00021% |
| **HTR fatality rate** | N/A | N/A | 10% |
| **Average nr of babies per (surviving) patient** | N/A | N/A | 0.10% |
| **Probability that baby is at risk of HDFN** | N/A | N/A | 2.34% |
| **Probability that an at risk baby dies of HDFN** | N/A | N/A | 0.015% |
| **Probability that an at risk baby has severe disability from HDFN** | N/A | N/A | 0.012% |
| **Post trauma life expectancy** | 48 | 48 | 48 |

References

1. Christensen MC, Banner C, Lefering R, Vallejo-Torres L, Morris S. Quality of life after severe trauma: results from the global trauma trial with recombinant Factor VII. *J Trauma* 2011; **70**(6): 1524-31.

2. Overgaard M, Hoyer CB, Christensen EF. Long-term survival and health-related quality of life 6 to 9 years after trauma. *J Trauma* 2011; **71**(2): 435-41.

3. Gravensteen IK, Helgadottir LB, Jacobsen EM, Sandset PM, Ekeberg O. Long-term impact of intrauterine fetal death on quality of life and depression: a case-control study. *BMC Pregnancy Childbirth* 2012; **12**: 43.

4. Pilgrim H, Lloyd-Jones M, Rees A. Routine antenatal anti-D prophylaxis for RhD-negative women: a systematic review and economic evaluation. *Health Technol Assess* 2009; **13**(10): iii, ix-xi, 1-103.

5. ONS. National Life Tables E, 1980-1982 to 2018-2020. *Available from: National life tables: UK - Office for National Statistics (onsgovuk)*.

6. Treasury. H. The Green Book: Central Government Guidance on Appraisal and Evaluation. *[Accessed at:* [*https://wwwgovuk/government/publications/the-green-book-appraisal-and-evaluation-in-central-governent/the-green-book-2020*](https://wwwgovuk/government/publications/the-green-book-appraisal-and-evaluation-in-central-governent/the-green-book-2020) 2020.
